# Microbiota dysbiosis impact on the metabolism of T3 and T4 hormones and its association with thyroid cancer

**DOI:** 10.3389/fcell.2025.1589726

**Published:** 2025-06-02

**Authors:** Santiago Cadena-Ullauri, Patricia Guevara-Ramírez, Elius Paz-Cruz, Viviana A. Ruiz-Pozo, Rafael Tamayo-Trujillo, Ana Karina Zambrano

**Affiliations:** Universidad UTE, Facultad de Ciencias de la Salud Eugenio Espejo, Centro de Investigación Genética y Genómica, Quito, Ecuador

**Keywords:** microbiota, thyroid, healthcare, cancer, dysbiosis, metabolism

## Abstract

This review explores the impact of gut microbiota dysbiosis on thyroid hormone metabolism and its potential association with thyroid cancer. The analysis highlights specific bacterial genera linked to thyroid dysfunction, the role of gut microbiota in iodine absorption, and mechanisms connecting dysbiosis with thyroid disorders such as hypothyroidism, hyperthyroidism, Hashimoto’s thyroiditis, and Graves’ disease. Additionally, it examines the potential of gut microbiota as a biomarker for diagnosis and personalized treatment, as well as the prospect of probiotics and microbiota-targeted treatments. The review emphasizes the importance of conducting additional research to fully understand microbiota-thyroid interactions and develop appropriate therapies to improve clinical outcomes and patient quality of life.

## Introduction

Thyroid cancer (TC) is the most common endocrine neoplasm, originating in the parenchymal cells of the thyroid. The thyroid parenchyma is comprised of two main cell types: thyroid follicular cells, which give rise to differentiated thyroid cancer (DTC), and parafollicular (C cells), which give rise to medullary thyroid carcinoma (MTC). DTC includes papillary thyroid cancer (PTC), follicular thyroid cancer (FTC), and Hürthle cell cancer, collectively accounts for 90%–95% of all thyroid cancers. Medullary thyroid carcinoma (MTC) accounts for approximately 1%–2%, while anaplastic thyroid carcinoma, the rarest and most aggressive form, represents less than 1% of all thyroid malignancies (Noone et al., 2016; Cadena-Ullauri et al., 2023; Paz-Cruz et al., 2023).

TC is the eighth most common cancer globally, with an incidence rate of 9.1 cases per 100,000 individuals. It also exhibits significant gender differences, ranking as the 14th most diagnosed cancer in men (4.6 cases per 100,000) and the fifth most diagnosed in women (13.6 cases per 100,000). Additionally, the number of diagnosed cases is projected to increase by 11.9% by 2030 (Bray Bsc et al., 2024).

The thyroid gland, located at the base of the neck, produces the thyroid hormones thyroxine (T4) and triiodothyronine (T3), which are essential for metabolic regulation, growth, and development. The synthesis of these hormones begins with the uptake of iodine by thyroid follicular cells, which then binds to thyroglobulin to form T3 and T4. Thyroid-stimulating hormone (TSH), released by the anterior pituitary gland, regulates the production and release of T3 and T4, which subsequently act on nearly all body tissues, influencing basal metabolism (Krashin et al., 2019).

Furthermore, the microbiota also plays a crucial role in endocrine control by modulating the hypothalamic-pituitary-adrenal (HPA) axis, which regulates the stress response and cortisol production, as well as thyroid hormone production. Additionally, the microbiota influences the synthesis of neurotransmitters like serotonin and short-chain fatty acids (SCFAs) such as butyrate, both of which affect insulin sensitivity and energy metabolism. Dysbiosis can contribute to various health issues, including metabolic disorders, chronic inflammation, autoimmune diseases, and cancer (Krashin et al., 2019; Rastelli et al., 2019; Pires et al., 2024).

This review explores the association between microbiota dysbiosis and thyroid hormone metabolism, and its implications for thyroid cancer. Furthermore, it analyzes the potential of microbiota as biomarkers for diagnosis and personalized treatment.

## Gut microbiota and thyroid hormone metabolism

Recent advancements in microbial research and microbiome assays have led to increased investigation of the relationship between gut microbiota and thyroid function, a connection referred to as the thyroid-gut axis (Lerner et al., 2017; Jiang et al., 2022). Notably, the gut microbiome has been associated with the regulation of endogenous and exogenous thyroid hormones (Bargiel et al., 2021; Fröhlich and Wahl, 2019). Moreover, Liu et al. (2023) identified specific genera, including *Intestimonas*, *Eubacterium brachy* groups, *Ruminiclostridium* 5 and Ruminococcaceae UCG-004, associated with an increased risk of thyroid dysfunction. In contrast, *Bifidobacterium*, Lachnospiraceae UCG-008, *Actinobacteria* and *Verrucomicrobia* were correlated with a protective role in maintaining thyroid function (Sessa et al., 2025; Liu et al., 2023).

### Role of gut microbiota in iodine absorption and thyroid function

The thyroid gland requires iodine, absorbed in the gastrointestinal tract, for hormone synthesis. Consequently, gut microbiota plays a crucial role in iodine metabolism and uptake (Jiang et al., 2022; Gong et al., 2024). However, the molecular mechanisms underlying this interaction have not been fully described. Studies suggest that lipopolysaccharides (LPS), components of the membranes of Gram-negative bacteria, can alter the expression of the sodium/iodine symporter (NIS), a key protein in iodine uptake, thereby disrupting thyroid function (Jiang et al., 2022; Knezevic et al., 2020; Nicola et al., 2010). Additionally, microbiota modulation has been shown to regulate iodine uptake, potentially reducing the dosage of levothyroxine (L-T4) in hypothyroidism patients (Jiang et al., 2022; Spaggiari et al., 2017).

SCFAs, metabolites produced by gut microbiota, have also been implicated in iodine absorption by influencing NIS activity (Jiang et al., 2022). For instance, butyrate can disrupt histone deacetylase (HDAC), leading to increased expression of NIS in TC cells. This upregulation induces re-differentiation and enhances iodine uptake (Zhou et al., 2018; Rathod et al., 2020).

### Microbial enzymes involved in T3 and T4 metabolism

Gut microbiota can directly influence the metabolism of T3 and T4 hormones. Iodothyronine deiodinases are enzymes responsible for regulating thyroid hormone synthesis, with three isoforms: type I (D1), type II (D2), and type III (D3) (Luongo et al., 2019). LPS can modulate the activity of these enzymes, particularly in the hypothalamus and anterior pituitary. LPS inhibits D1, while activating D2, leading to an enhanced conversion of T4 to T3 (Jiang et al., 2022; Fekete et al., 2004).

### Role of gut barrier integrity: immune modulation and inflammation

The intestinal epithelial lining serves a barrier that prevents harmful compounds, such as toxins, and microorganisms, from entering the body (Ramanan and Cadwell, 2016). When this barrier is compromised, increased permeability allows the entry of these pathogens into systemic circulation, potentially triggering immune dysregulation (Sessa et al., 2025). For instance, certain bacterial genera such as *Lactobacilli* and *Bifidobacteria*, share protein sequence similarities with thyroglobulin and thyroid peroxidase, key thyroid proteins. As a result, they may contribute to autoimmune reactivity via molecular mimicry mechanisms, thereby influencing thyroid hormone production and potentially leading to thyroid dysfunction (Sessa et al., 2025). Additionally, dysbiosis may influence the gut-brain-thyroid axis by altering receptor signaling, potentially affecting neuroendocrine regulation and thyroid function (Fang and Ning, 2024; Zhu et al., 2024).

## Microbiota dysbiosis and thyroid dysfunction

### Evidence linking gut microbiota alterations with hypothyroidism and hyperthyroidism

The intestinal microbiota influences thyroid hormone metabolism (T3 and T4) through micronutrient absorption, microbial enzymes, and immune cell interactions (Jiang et al., 2022). Evidence suggests that microbiota alterations may contribute to thyroid disorders like hypothyroidism and hyperthyroidism.

Hypothyroidism, characterized by impaired thyroid function and reduced hormone production, has been linked to specific gut microbiota changes. A study comparing 52 patients with primary hypothyroidism to 40 healthy controls found a significant decrease in the genera *Veillonella, Paraprevotella, Neisseria,* and *Rheinheimera*. Additionally, hypothyroid patients exhibited reduced production of SCFAs, leading to elevated serum LPS levels, which contribute to microbiota dysbiosis and disrupted metabolic and immune signaling (Su et al., 2020). Although these specific genera are not directed SCAF producer, their depletion may reflect broader community dysbiosis that impairs butyrate availability. Moreover, elevated LPS (likely produced by Gram-Negative bacteria), can inhibit D1, reducing peripheral conversion of T4 to T3, while upregulating type D2 in hypothalamus, thereby altering systemic thyroid hormone (Jiang et al., 2022; Fekete et al., 2004). *Intestinimonas*, and *Ruminiclostridium 5* (Gram-negative bacteria) were associated with an increased risk of hypothyroidism (Shi et al., 2024), may thus contribute mechanistically by promoting inflammatory signals that disrupt hormonal balance.

Another study conducted by Shi et al. (2024) used Mendelian randomization (MR) analysis to evaluate the causal relationship between intestinal microbiota and hypothyroidism. The results suggested that the genera *Akkermansia*, *Holdemania*, *Butyrivibrio*, and Ruminococcaceae UCG-011 may have a protective effect against hypothyroidism; hypothyroidism (Shi et al., 2024). The genera *Butyrivibrio*, and Ruminococcaceae UCG-011 are known producers of SCFAs such as butyrate, which enhances NIS expression via inhibition of HDACs. This epigenetic modulation increases iodine uptake by thyroid follicular cells, supporting optimal T3 and T4 biosynthesis (Zhou et al., 2018; Rathod et al., 2020; Shi et al., 2024).

Hyperthyroidism, caused by inhibition of thyrotropin (TSH) production and elevated T3 levels (Lee and Pearce, 2023) has also been linked to gut microbiota dysregulation. Research by Xie et al. (2023) analyzed genome-wide association data (GWAS) from the FinnGen consortium, including 1,621 cases of hyperthyroidism and 255,931 controls. Their findings identified a positive association between hyperthyroidism risk and the genera Ruminococcaceae*, Prevotella 7, Collinsella, Catenibacterium,* and *Bilophila*. Among these *Bilophila* and *Collinsella* are linked to increased production of LPS and proinflammatory metabolites, promoting systemic inflammation, impair deiodinase activity, and alter TSH regulation. Furthermore, reduced abundance of *Deltaproteobacteria* was associated with a lower hypothyroidism risk (Xie et al., 2023).

Another study observed a significant decrease in *Bifidobacterium* and *Lactobacillus*, along with an increase in *Enterococcus* in individuals with hyperthyroidism suggesting a microbial imbalance that may contribute to disease progression (Zhou et al., 2014). The loss of *Bifidobacterium* and *Lactobacillus* compromises SCFA production and intestinal barrier integrity, reducing NIS expression and impairing iodine absorption (Jiang et al., 2022; Fekete et al., 2004).

### Potential mechanisms linking dysbiosis with abnormal thyroid function

The intestinal microbiota regulates immune balance, influencing inflammatory responses. Dysbiosis has been implicated in the pathogenesis of autoimmune thyroid diseases, including Hashimoto’s thyroiditis (HT) and Graves’ disease (GD) (Virili et al., 2023).

HT is an autoimmune disorder characterized by chronic inflammation and the presence of autoantibodies against thyroid peroxidase (TPO) and thyroglobulin, which contribute to progressive thyroid dysfunction. Zhao et al. (2018) analyzed the gut microbiota of HT patients and identified dysbiosis correlated with clinical parameters of the disease (Zhao et al., 2018). Furthermore, a study comparing 40 HT patients with 53 healthy controls reported an increase in abundance of *Bacteroides* species and a decrease in *Bifidobacterium* species. *Bacteroides* are potent LPS producers capable of activating TLR4 in thyroid follicular cells (Mancuso et al., 2005), while Some strains of *Bifidobacterium* are involved in the maturation of the immune system and metabolism (Cayres et al., 2021).

In contrast, GD is associated with an increase in *Prevotella* and *Bacteroides*, which are linked to the activation of autoreactive T cells and the production of thyroid-stimulating antibodies (TSAbs). This immune imbalance results in continuous thyroid stimulation, leading to both excessive hormone production and glandular hyperplasia, characteristic of GD (Chen et al., 2024; Sawicka-Gutaj et al., 2022; Liu H. et al., 2022).

## Microbiota dysbiosis and thyroid cancer: potential pathways

### Chronic inflammation and oxidative stress

#### Gut-derived endotoxins and systemic inflammation

The gut microbiota maintains immune homeostasis and metabolic balance (Guevara-Ramírez et al., 2024). Dysbiosis can lead to an accumulation of endotoxins and exotoxins, inducing DNA damage or genetic instability, which promote tumorigenesis and cancer progression (Liu Q. et al., 2022). For instance, LPS can modify thyroid-specific gene expression (Vélez et al., 2006; Nicola et al., 2009).

Gut microbiota dysbiosis can trigger immune responses by activating Toll-like receptors (TLRs) and stimulating the excessive production of pro-inflammatory cytokines, including tumor necrosis factor-α, interleukin-1β, and interferon-γ. TLR4 recognizes LPS through a mechanism involving Cluster of Differentiation 14 (CD14) and myeloid differentiation factor 2 (MD2) (Miyake, 2004; Kim and Kim, 2017). CD14 facilitates LPS transfer to the TLR4/MD2 complex, leading to activation of intracellular signaling cascades via MyD88, TIRAP, TRIF, and TRAM. This cascade triggers nuclear factor-kappa B (NF-κB), activator protein-1 (AP-1), and interferon regulatory factors, inducing immune response gene expression. However, excessive LPS-driven responses can cause systemic inflammation, leading to the development of autoimmune thyroid diseases (AITDs) (Sessa et al., 2025; Kamada et al., 2013; Belkaid and Hand, 2014), tissue damage, organ failure, and even septic shock (Kim and Kim, 2017). In this context, recent studies using 16S rRNA sequencing have identified a correlation between gut microbiota dysbiosis and the severity of AITDs (Yan et al., 2024; Alkader et al., 2023).

Moreover, oxidative stress results from elevated reactive oxygen species (ROS), influences the tumor microenvironment by promoting angiogenesis, metastasis and survival (Mittal et al., 2014). If ROS levels remain low, therapy may activate NF-κB, PI3K, HIFs and MAPKs pathways, fostering tumorigenesis. Conversely, increased ROS levels can induce cancer cell death (Schieber and Chandel, 2014).

#### Role of inflammatory cytokines in thyroid carcinogenesis

The inflammatory microenvironment contributes cancer initiation, progression and metastasis. Interferons (IFNs), particularly IFN-γ and TNF-α, have been linked to tumor malignancy, inducing epithelial-to-mesenchymal transition in PTC cells (Lv et al., 2015; Zhang et al., 2018).

Furthermore, IL-6, acting as both a pro-inflammatory cytokine and anti-inflammatory myokine, influences tumor progression across cancer types. The IL-6/JAK2/STAT3 pathway upregulates DNA methyltransferase 1, promoting tumor progression. Similarly, IL-17 facilitates cancer progression (Zhang et al., 2018).

### Epigenetic modifications and microbiota influence

#### Microbial metabolites affecting DNA methylation and histone modifications

Gut microbiota metabolites such as acetate, propionate and butyrate, influence epigenetic modifications, and constitute 95% of the total SCFAs (Krautkramer et al., 2021). Butyrate has been linked to histone acetylation and potential cancer promotion (Krautkramer et al., 2021). Additionally, bacterial-derived folate and methionine contribute to DNA methylation stability. Alterations in these metabolic pathways may dysregulate inflammatory and oncogenic gene expression linking to carcinogenesis (Kok et al., 2018; Crider et al., 2012).

#### Impact on oncogenes and tumor suppressor genes in thyroid cancer

There are no reports of a direct association between gut microbiota dysbiosis and oncogene activation or tumor suppressor gene inactivation. However, a clear association between chronic inflammation and cancer development has been established (Zhao et al., 2021). Therefore, gut microbial dysbiosis could promote intestinal inflammation and bacterial invasion in a gut-thyroid axis form (Gorini and Tonacci, 2023; Zhang et al., 2019). Once the thyroid tissue has been colonized, an immune response could start with the recognition of pathogen-associated molecular patterns (PAMPs) by macrophages through TLR signaling, which promotes the release of pro-inflammatory cytokines. These cytokines promote the activation of nuclear factor-κB, which promotes the tissue release of TNF, IL-1, IL-6, growth factors, and anti-apoptotic proteins (Taniguchi and Karin, 2018). It has been suggested that chronic inflammation could promote oncogene mutation and activation, along with tumor suppressor gene inactivity (Zhao et al., 2021; Coussens and Werb, 2002; Crescenzi et al., 2024). Therefore, this inflammatory process could promote the activation and/or mutation of several oncogenes (*BRAF*, *RAS*) and tumor suppressor genes (*PTEN*, *PPARγ*, *TP53*) in thyroid tissue, which could initiate oncogenesis.

### Gut microbiota-driven metabolic shifts favoring tumorigenesis

#### Dysbiosis-induced hormonal imbalances

Several studies have reported that gut microbiota influences hormonal indices such as TSH, FT4 (Free tetraiodothyronine), and FT3 (Free triiodothyronine). The microbiota may modulate thyroid function through multiple mechanisms: by facilitating the conversion of T4 into T3; by disrupting the uptake of micronutrients like iodine, selenium, or zinc, which are necessary for thyroid hormone biosynthesis; by promoting amino acid degradation, or in case of dysbiosis, leading to a decrease butyrate production (Liu Q. et al., 2022; Yuan et al., 2022).

Research has also demonstrated a correlation between specific intratumoral bacterial genera and thyroid hormone synthesis. For example, a positive association was found between FT4 levels and the presence of *Neisseria* (Yuan et al., 2022). Similarly, an increased abundance of *Porphyromonas* and *Streptococcus* has been positively linked to increased synthesis of TSH and FT3 in TC patients (Zhang et al., 2019).

Notably, patients with thyroid nodules have exhibited reduced levels of butyrate-producing bacteria (*Butyrivibrio*, *Coprococcus catus*, *Roseburia hominis*, *Eubacterium eligens*), as well as a marked absence of SCFA-producing bacteria (*Butyricimonas*) (Li et al., 2021). SCFAs may be involved in immune function processes, such as inducing antimicrobial activity and inhibiting cytokine production (Gorini and Tonacci, 2023; Helmink et al., 2019). Therefore, a deficiency of SCFA-producing bacteria may impair immune responses following microbial colonization in thyroid tissue, potentially triggering chronic inflammation and oncogenesis.

**FIGURE 1:**
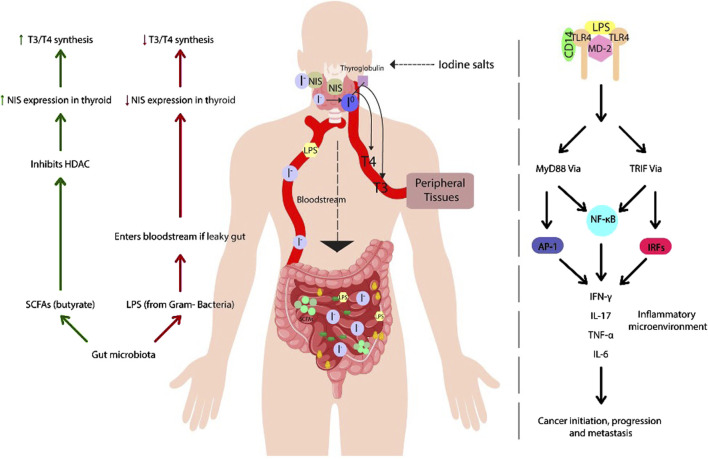
Effects of gut microbiota on thyroid regulation: role of SCFAs and LPS in hormone homeostasis and inflammation: the figure illustrates the interaction between gut microbiota and thyroid function, highlighting beneficial and detrimental mechanisms regulating hormone homeostasis. Short-chain fatty acids (SCFAs), such as butyrate, play a key role in modulating the expression of the sodium-iodine cotransporter (NIS), promoting iodine uptake and synthesis of the thyroid hormones T3 and T4. However, intestinal dysbiosis can disrupt this balance, increasing intestinal permeability and allowing lipopolysaccharide (LPS) to enter the bloodstream, which inhibits NIS expression. Furthermore, LPS recognition by Toll-like receptor 4 (TLR4) activates intracellular signaling cascades that promote the release of proinflammatory cytokines, potentially contributing to the development of thyroid autoimmune diseases and tumors.

Furthermore, the intratumoral microbiome has been shown to be metabolically active, influencing key cellular processes through its secreted metabolites. These interactions can affect cancer progression, metastasis, and inflammation by interacting with signaling pathways such as MAP/ERK signaling pathway, which involve oncogenic proteins like BRAF and RAS (Dai et al., 2021; Poulikakos et al., 2022). Thus, microbiota-secreted metabolites may play a critical role in cancer-associated molecular processes (Figure 1).

Despite these associations, the role of thyroid hormones in cancer initiation, progression, and metastasis remains controversial. Some studies suggest that elevated thyroid hormone levels and hyperthyroidism are associated with increased tumor growth and malignancy (Lasa and Contreras-Jurado, 2022; Zhang et al., 2019; Kim et al., 2019; Tran et al., 2020). Conversely, other findings indicate that hypothyroidism and decreased hormone levels may favor tumor spread and increased cancer predisposition (Lasa and Contreras-Jurado, 2022; Tseng et al., 2015). These conflicting results highlight the need for further research to clarify the intricate relationship between thyroid function and cancer biology.

## Clinical and translational perspectives

### Microbiota-based biomarkers for thyroid cancer risk assessment

In recent decades, the microbiota has been widely recognized for its role in the carcinogenesis of various types of cancer, especially the intestinal and oral microbiota (Kun et al., 2023). Moreover, the finding of microbial communities in organs, historically considered sterile, such as the thyroid gland, has opened new perspectives in oncology (Xie et al., 2024). Recent studies have demonstrated the presence of a diverse microbial environment in thyroid tissue, suggesting microbiota as a potential biomarker for TC development (Yuan et al., 2022; Yu et al., 2023).

Comparative studies have shown that the microbial composition of thyroid tissue differs significantly between malignant and benign tumors, and even across cancer stages. For example, species like *Sphingomonas* have shown strong potential as biomarkers, with higher abundance in more advanced stages (N1) compared to stage N0 (Dai et al., 2021). Likewise, tumor microbiota diversity is significantly higher in patients with advanced stages (T3/T4) than those with early-stage lesions (T1/T2). Additionally, women tend to show greater microbial diversity than men, which could be linked to their increased susceptibility to TC (Xie et al., 2024).

Similarly, the oral microbiota has shown significant alterations in patients with TC. These changes may be caused by altered levels of thyroid hormones, especially TSH. There is evidence that certain oral bacteria may enter the gastrointestinal tract, contributing to dysbiosis in these patients. Studies have linked high TSH levels with an increase in *Porphyromonas* (Lin et al., 2022a), *Alloprevotella*, *Anaeroglobus*, and *Acinetobacter* (Kun et al., 2023). Additionally, FT3 is positively related to *Streptococcus* and negatively related to *Lactobacillus*. *Lactobacillus* may help control inflammation through the production of SCFAs, highlighting the role of the microbiota in disease and its potential use in diagnosis and prognosis (Zhang et al., 2019).

### Probiotics, prebiotics, and microbiota-targeted therapies

In recent years, probiotics and prebiotics have been important for healthy eating and as supplements to improve health (Yu et al., 2022). Although direct evidence in TC is limited, studies in hypothyroidism and Graves’ disease suggest that they may improve thyroid function, especially in combination with drugs (Talebi et al., 2020;Han et al., 2022; Huo et al., 2021).

Talebi et al. (2019) conducted a randomized, double-blind, placebo-controlled clinical trial from November 2018 to February 2019, enrolling 60 adults (18–65 years) with primary hypothyroidism under stable LT4 therapy. Participants were randomly assigned to receive either 500 mg/day of a symbiotic supplement (n = 30) or a placebo (n = 30) for 8 weeks. The symbiotic supplement (Familact) contained seven probiotic strains: *Lactobacillus casei* (7 × 10^9^ CFU), *Lactobacillus acidophilus* (2 × 10^9^ CFU), *Lactobacillus rhamnosus* (1.5 × 10^9^ CFU), *Lactobacillus bulgaricus* (2 × 10^8^ CFU), *Bifidobacterium breve* (2 × 10^10^ CFU), *Bifidobacterium longum* (7 × 10^9^ CFU), and *Streptococcus thermophilus* (1.5 × 10^10^ CFU), along with a prebiotic (fructooligosaccharides). Supplements were administered once daily, 2 h after LT4 (post-breakfast), with no change in LT4 dosage. The symbiotic group demonstrated significant improvements in thyroid function, including reductions in TSH levels (P = 0.007) and LT4 dosage (P = 0.043), as well as increases in FT3 levels (P = 0.000) and a decreased FT3/TSH ratio (P = 0.000). In contrast, the placebo group exhibited a significant increase only in FT3 levels (P = 0.000). However, between-group comparisons—adjusted for baseline values, type of hypothyroidism, and body mass index (BMI)—revealed no statistically significant differences in TSH (P = 0.605), FT3 (P = 0.490), FT3/TSH ratio (P = 0.164), or LT4 dosage (P = 0.120) (Talebi et al., 2020), pointing out that further studies with larger sample sizes and longer follow-up periods are needed to confirm and expand upon these preliminary results.

Similarly, two 6-month clinical trials investigated the effects of gut microbiota modulation on thyroid function in patients with Graves’ disease treated with methimazole (MI). In the first study, patients were assigned to three groups: MI alone (n = 8), MI plus black bean (n = 9), and MI plus the probiotic Bifidobacterium longum (n = 9) (Huo et al., 2021). On the other hand, the second study compared MI alone (20 mg/day, n = 8) with MI plus the prebiotic berberine (0.9 g/day in three doses, n = 10) (Han et al., 2022). Both studies, despite evaluating different co-interventions converge on the limitations of standard methimazole therapy for Graves’ disease, particularly its insufficient effect on normalizing TRAb levels and its disruptive impact on the gut microbiota. The findings suggest that modulating the intestinal microbiota, whether through probiotic or prebiotic supplementation, may represent a promising strategy to enhance treatment outcomes in patients with Graves’ disease (Han et al., 2022; Huo et al., 2021). However, a recent meta-analysis indicates that their effects on thyroid function and other parameters are inconsistent, highlighting the need for further research (Zawadzka et al., 2023).

In thyroid cancer (TC), although the available evidence is more limited, the gut microbiota appears to play a relevant role. Lin et al. (2022a) conducted a randomized, double-blind, placebo-controlled, parallel-group clinical trial involving 50 post-thyroidectomy patients with differentiated thyroid cancer (DTC) undergoing thyroid hormone withdrawal (THW). Participants were randomly assigned to receive either a probiotic formulation or a placebo for 4 weeks. The probiotic consisted of Bifidobacterium infantis, *Lactobacillus* acidophilus, *Enterococcus faecalis*, and *Bacillus* cereus, administered as three capsules twice daily.

In TC, although the evidence is more limited, the microbiota seems to play a relevant role. Lin et al. (2022) conducted a randomized, double-blind, placebo-controlled, parallel-group clinical trial in 50 post-thyroidectomy patients with differentiated thyroid cancer (DTC) undergoing thyroid hormone withdrawal (THW). Participants were randomly assigned to receive either a probiotic formulation or a placebo for 4 weeks. The probiotic included *Bifidobacterium infantis*, *L. acidophilus*, *E. faecalis*, and *Bacillus cereus*, administered as three capsules twice daily (Lin et al., 2022b).

In the placebo group, THW significantly reduced microbial diversity in both the gut and oral microbiota, increased the microbial dysbiosis index (MDI), elevated lipopolysaccharide (LPS) levels in stool and plasma, and worsened lipid profiles, including total cholesterol, triglycerides, LDL, and Apo A. In contrast, probiotic supplementation significantly restored microbial diversity, reduced MDI, and modulated microbiota composition by increasing the abundance of beneficial genera such as *Holdemanella*, *Enterococcus*, and *Coprococcus_2*, while decreasing potentially harmful genera including *Fusobacterium*, *Eubacterium_ruminantium_group*, and *Parasutterella* in the gut, and *Prevotella_9*, *Haemophilus*, *Fusobacterium*, and *Lautropia* in the oral cavity (Lin et al., 2022b).

Clinically, probiotic administration was associated with a reduction in both the incidence and severity of THW-related complications, as well as significant improvements in lipid profiles. Furthermore, LPS levels in both stool and plasma were markedly reduced. Thyroid function markers (fT3, fT4, TSH, and thyroglobulin) remained unchanged between groups, although a non-significant trend toward increased fT3 levels was observed in the probiotic group (Lin et al., 2022b). This suggests that in oncology, modifying the microbiota could improve therapeutic outcomes, bringing new perspectives to the comprehensive treatment of TC (Yu et al., 2022; Talebi et al., 2020; Huo et al., 2021; Han et al., 2022; Zawadzka et al., 2023; Lin et al., 2022b).

### Future directions in personalized medicine

The role of the microbiota in TC presents new opportunities for prevention and treatment. However, large-scale longitudinal and metagenomic studies are needed to understand microbial dynamics across disease stages and interactions with genetic and environmental factors (Gorini and Tonacci, 2023). By integrating metagenomics, transcriptomics, and epigenetics, researchers could identify predictive biomarkers. Additionally, interventions that modify the microbiota, such as probiotics, prebiotics, and fecal transplantation, show potential for restoring balance and improving treatment responses (Fröhlich and Wahl, 2019). This multidisciplinary approach may enable personalized therapies, improving clinical outcomes and quality of life for TC patients.

## Conclusion

This review highlights the significant role of gut microbiota in thyroid hormone metabolism and its impact on thyroid diseases, including cancer. Key findings suggest that specific microbial signatures could serve as biomarkers for diagnosis and personalized treatment. Probiotics and microbiota-targeted therapies show promise in improving thyroid function and therapeutic outcomes. However, further research is needed to fully understand microbiota-thyroid interactions and develop effective interventions, emphasizing the importance of integrating metagenomics, transcriptomics, and epigenetics in future studies to enhance clinical outcomes and patient quality of life.
